# Factors Associated with Progression to SCAI-Defined Hemodynamic Instability at 72 Hours in Patients with Acute Decompensated Heart Failure Receiving Inotropic Therapy

**DOI:** 10.3390/jcm15155816

**Published:** 2026-07-25

**Authors:** Rauf Avcı, Gülsüm Meral Yılmaz Öztekin

**Affiliations:** Turkish Ministry of Health Antalya Training and Research Hospital, 07100 Antalya, Turkey

**Keywords:** acute decompensated heart failure, inotropic therapy, SCAI shock classification, hemodynamic instability, diastolic blood pressure, cardiogenic shock, white blood cell count

## Abstract

**Background:** Early hemodynamic deterioration may occur in patients with acute decompensated heart failure (ADHF) receiving inotropic therapy despite the absence of overt hypoperfusion at baseline. This study aimed to identify baseline factors associated with progression from Society for Cardiovascular Angiography and Interventions (SCAI) stage A or B to SCAI stage C–E at 72 h after initiation of inotropic therapy. **Methods:** This retrospective, single-center study included 375 adults with ADHF who received intravenous dobutamine or levosimendan and were classified as SCAI stage A or B at the time of inotrope initiation. The primary endpoint was progression to SCAI stage C, D, or E at the 72 h assessment. Baseline clinical, echocardiographic, laboratory, and hemodynamic variables were compared according to progression status. Univariable and multivariable logistic regression analyses were performed to identify factors associated with the primary endpoint. **Results:** Progression to SCAI stage C–E occurred in 30 patients (8.0%). Patients who progressed had lower diastolic blood pressure (DBP) and higher heart rate, white blood cell count (WBC), C-reactive protein, aspartate aminotransferase, lactate dehydrogenase, and N-terminal pro-B-type natriuretic peptide levels. The primary multivariable logistic regression model included 327 patients and all 30 outcome events. Higher WBC was independently associated with greater odds of progression (odds ratio: 1.099; 95% confidence interval: 1.030–1.174; *p* = 0.005), whereas higher DBP was independently associated with lower odds of progression to SCAI stage C–E (odds ratio: 0.963; 95% confidence interval: 0.934–0.992; *p* = 0.014). The model showed acceptable calibration and moderate discrimination, with a c-statistic of 0.731 (95% confidence interval: 0.630–0.832). In bootstrap analysis, the association remained significant for WBC, whereas the association with DBP was borderline. **Conclusions**: Higher WBC and lower DBP were associated with progression to SCAI stage C–E at 72 h in this exploratory single-center cohort of patients with ADHF receiving inotropic therapy.

## 1. Introduction

Acute heart failure (AHF) is a serious clinical syndrome characterized by the rapid onset of symptoms and signs of heart failure (HF) or the rapid worsening of pre-existing HF, frequently requiring hospitalization [1]. In patients with acute decompensated heart failure (ADHF), progressive hemodynamic deterioration and the subsequent development of end-organ hypoperfusion may lead to cardiogenic shock and adverse clinical outcomes.

Inotropic therapies are commonly used in patients with AHF to improve reduced cardiac output and maintain systemic perfusion [2]. However, determining which patients should receive inotropic support and when it should be initiated remains one of the most critical decisions in clinical practice.

The clinical course after initiation of inotropic therapy is heterogeneous, and some patients may deteriorate despite the absence of overt hypoperfusion at baseline. Early identification of patients at risk of progression to more advanced hemodynamic compromise may therefore facilitate closer monitoring and timely escalation of treatment.

The Society for Cardiovascular Angiography and Interventions (SCAI) shock classification provides a practical framework for bedside assessment and serial monitoring of cardiogenic shock severity [3]. This classification system allows cardiogenic shock to be assessed as a dynamic clinical process and has shown a strong association with mortality across different clinical populations. Previous studies have also demonstrated that early progression in SCAI stage is associated with adverse clinical outcomes and increased mortality [4,5,6,7].

However, baseline factors associated with early progression to SCAI stages C–E have not been sufficiently investigated in patients hospitalized with ADHF who receive inotropic therapy while initially classified as SCAI stage A or B. Although the individual prognostic variables examined are clinically familiar, evidence regarding their association with early SCAI stage progression in this specific high-risk population remains limited. Therefore, the present study aimed to identify baseline clinical, laboratory, echocardiographic, and hemodynamic factors associated with progression from SCAI stage A or B at baseline to SCAI stage C, D, or E at 72 h after initiation of inotropic therapy.

## 2. Materials and Methods

### 2.1. Study Design and Patient Population

This retrospective, single-center observational study evaluated patients who were followed in the coronary intensive care unit (CICU) for ADHF and received intravenous inotropic therapy between January 2015 and June 2025. Clinical, laboratory, echocardiographic, and treatment-related data were retrospectively obtained from the hospital electronic medical record system, archived patient files, and outpatient clinic records. Baseline laboratory values were defined as measurements obtained before initiation of inotropic therapy or, when unavailable, from the sample obtained closest to the time of inotrope initiation. No formal adjustment was made for fluid overload or hemodilution.

The diagnosis of ADHF was established according to contemporary guideline recommendations based on the integrated assessment of clinical findings, laboratory parameters, and cardiac imaging [8].

Pulmonary hypertension-associated isolated right-sided HF was considered a clinical phenotype rather than an etiologic category. This phenotype was defined by clinical and echocardiographic evidence of predominant right ventricular failure associated with pulmonary hypertension, without evidence of left-sided HF. Because the underlying cause and World Health Organization group of pulmonary hypertension could not be reliably determined from the retrospective records, pulmonary hypertension was not further classified by etiology. HF etiology was classified separately as ischemic, non-ischemic, or unknown according to the available clinical records.

Patients aged ≥18 years who were admitted to the CICU, received intravenous dobutamine or levosimendan therapy, and were classified as SCAI stage A or B at the time of inotrope initiation were eligible for inclusion.

This retrospective study evaluated a selected high-risk population in whom intravenous inotropic therapy had already been initiated in routine clinical practice based on the attending physician’s overall clinical and hemodynamic assessment. In patients classified as SCAI stage A, treatment was not protocol-driven or initiated solely on the basis of SCAI stage, but reflected individualized clinical decisions in the setting of advanced ADHF, inadequate response to standard therapy, persistent congestion, suspected low-output physiology, or concern for imminent hemodynamic deterioration.

Patients were considered to have no overt hypoperfusion at baseline in the absence of clear clinical signs of end-organ hypoperfusion, including altered mental status, cold or mottled extremities, oliguria, or other findings consistent with impaired peripheral perfusion. Lactate measurements were reviewed when available; however, lactate was not routinely available in all patients and was therefore not used as a mandatory criterion for baseline classification.

Admission to the CICU was based on guideline-recommended criteria for severe hemodynamic or respiratory deterioration, including oxygen saturation <90% despite oxygen therapy, use of accessory respiratory muscles, heart rate <40 or >130 beats/min, or systolic blood pressure (SBP) <90 mmHg [9].

Patients classified as SCAI stage C, D, or E at baseline were excluded. Additional exclusion criteria were mechanical complications of acute myocardial infarction, active infection or septic shock, use of temporary mechanical circulatory support at baseline, severe primary valvular heart disease requiring urgent intervention, active malignancy, age <18 years, and missing data required for baseline SCAI classification or primary endpoint assessment.

### 2.2. Inotropic Treatment Protocol

At our center, intravenous inotropic therapy was initiated in selected patients with ADHF on the basis of the attending physician’s overall clinical and hemodynamic assessment. Treatment decisions were individualized and took into account blood pressure, heart rate and rhythm, urine output, renal and hepatic function, congestion burden, suspected low cardiac output physiology, inadequate response to standard medical therapy, and the perceived risk of impending hemodynamic deterioration. The choice of inotropic agent was not protocol-mandated and reflected the patient’s clinical profile, contraindications, institutional practice, and physician judgment.

Dobutamine was generally initiated at a dose of 2.5–5 µg/kg/min and titrated according to the patient’s hemodynamic response, including blood pressure, heart rate, rhythm status, urine output, and peripheral perfusion, up to 20 µg/kg/min when clinically required.

Levosimendan was administered as a continuous intravenous infusion, generally at a dose of 0.05–0.2 µg/kg/min. A loading dose was not routinely administered, particularly in patients with low blood pressure or an increased risk of hypotension. The infusion rate was adjusted according to blood pressure, rhythm status, and clinical response.

Vasopressor therapy, primarily norepinephrine, was added when hypotension persisted or developed despite inotropic support, particularly when maintenance of adequate arterial pressure and organ perfusion was considered necessary. Escalation to multiple inotropes and/or vasopressors was based on serial clinical and hemodynamic reassessment and was considered a post-baseline treatment variable.

### 2.3. SCAI Shock Classification and Endpoint

Hemodynamic instability and shock severity were assessed according to the SCAI shock classification [3]. Baseline SCAI stage was assigned at the time of initiation of intravenous inotropic therapy, and patients were reassessed 72 h later using the clinical, hemodynamic, laboratory, and treatment-related variables available at each time point.

SCAI stage A was defined as a high-risk clinical state without hypotension or evidence of hypoperfusion. SCAI stage B was defined by the presence of early hemodynamic instability, such as hypotension or compensatory tachycardia, without overt clinical or biochemical evidence of end-organ hypoperfusion [3]. Progression to SCAI stages C–E was determined by the development of hypoperfusion and increasing shock severity, based on blood pressure, heart rate, mental status, peripheral perfusion, urine output, renal and hepatic function, available lactate measurements, and the intensity of vasoactive or mechanical circulatory support. Lactate values were incorporated when available but were not required as a mandatory criterion because they were not uniformly measured in all patients.

The primary endpoint was progression from SCAI stage A or B at baseline to SCAI stage C, D, or E at the 72 h assessment after initiation of inotropic therapy. Patients who remained in or transitioned between SCAI stages A and B at 72 h were classified as having no progression to the primary endpoint.

Because escalation of vasoactive therapy may contribute to SCAI staging, post-baseline use of additional inotropes or vasopressors was considered part of the clinical trajectory and was not included as a baseline predictor in the multivariable regression model.

### 2.4. Statistical Analysis

Statistical analyses were performed using IBM SPSS Statistics, version 26.0 (IBM Corp., Armonk, NY, USA). The distribution of continuous variables was assessed using the Kolmogorov–Smirnov test together with visual inspection. Continuous variables are presented as mean ± standard deviation or median with interquartile range (IQR), as appropriate, whereas categorical variables are presented as numbers and percentages. Between-group comparisons of continuous variables were performed using Student’s *t*-test or the Mann–Whitney *U* test, according to data distribution. Categorical variables were compared using the chi-square test or Fisher’s exact test, as appropriate.

Patients were classified according to the SCAI assessment performed 72 h after initiation of inotropic therapy. The primary endpoint was defined as progression from baseline SCAI stage A or B to SCAI stage C, D, or E at the 72 h assessment.

Associations between baseline variables and progression to SCAI stage C–E at 72 h were first evaluated using univariable logistic regression analyses. Because aspartate aminotransferase (AST), lactate dehydrogenase (LDH), and N-terminal pro-B-type natriuretic peptide (NT-proBNP) showed markedly right-skewed distributions, these variables were natural logarithm-transformed before regression analysis. Results are reported as odds ratios (ORs) with 95% confidence intervals (CIs) and *p*-values.

Candidate variables for the multivariable logistic regression model were selected on the basis of clinical relevance, univariable associations, and potential overlap among variables representing the same clinical domain. Spearman correlation analysis was used to assess relationships among variables that were significant in univariable analyses. A weak-to-moderate positive correlation was observed between C-reactive protein (CRP) and white blood cell (WBC) count, a moderate positive correlation between AST and LDH, and only a very weak correlation between diastolic blood pressure (DBP) and heart rate.

To avoid including variables representing overlapping clinical domains and to reduce the risk of overfitting, WBC was selected as the inflammatory marker and DBP as the hemodynamic marker for inclusion in the primary multivariable model. Although chronic obstructive pulmonary disease (COPD) was associated with progression in univariable analysis, it was not included because of the limited number of outcome events and the need to maintain a parsimonious model. Because NT-proBNP measurements were available only in a subset of the cohort, natural logarithm-transformed NT-proBNP [ln(NT-proBNP)] was also excluded from the primary model. Post-baseline treatment-escalation variables were not included because they could represent components of the clinical pathway leading to the outcome.

Model calibration was assessed using the Hosmer–Lemeshow goodness-of-fit test, model explanatory value was reported using the Nagelkerke R^2^, and model discrimination was evaluated using the area under the receiver operating characteristic curve, expressed as the c-statistic. Internal validation and coefficient stability were assessed using bootstrap resampling with 2000 samples. No imputation was performed for missing data, and analyses were conducted using available cases. All statistical tests were two-sided, and a *p*-value of <0.05 was considered statistically significant.

## 3. Results

**Figure 1:**
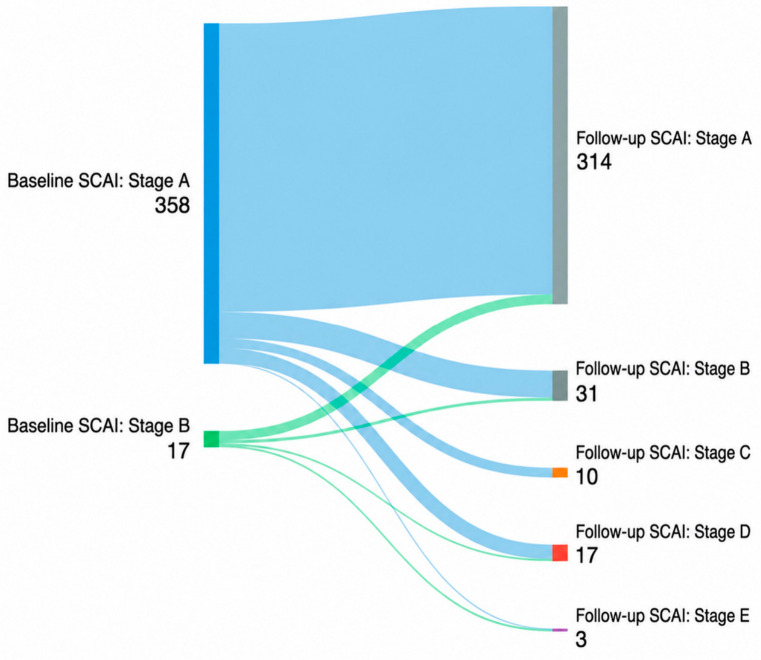
Transitions from baseline SCAI stages A and B to SCAI stages A–E at 72 h after initiation of inotropic therapy. Blue and green flow bands represent transitions originating from baseline SCAI stages A and B, respectively, while the colored terminal bars indicate the corresponding SCAI stages at 72 h. Abbreviation: SCAI, Society for Cardiovascular Angiography and Interventions.

A total of 375 patients with ADHF who received intravenous inotropic therapy were included. At baseline, 358 patients (95.5%) were classified as SCAI stage A and 17 patients (4.5%) as SCAI stage B. At 72 h after initiation of inotropic therapy, 30 patients (8.0%) had progressed to SCAI stage C, D, or E, whereas 345 patients (92.0%) remained in SCAI stage A or B. Transitions in SCAI stage from baseline to 72 h after initiation of inotropic therapy are illustrated in Figure 1.

Baseline characteristics according to progression status are presented in Table 1. The median age of the overall cohort was 69 years (IQR, 60–79), and 278 patients (74.1%) were male. Age, sex, and most comorbidities were comparable between groups. Chronic obstructive pulmonary disease (COPD) was more frequent among patients who progressed to SCAI stage C–E than among those who did not [8 (26.7%) vs. 31 (9.0%), Fisher’s exact *p* = 0.007]. HF etiology, HF phenotype, echocardiographic parameters, and the prevalence of significant valvular disease did not differ significantly between groups.

Patients who progressed to SCAI stage C–E had higher WBCs [11.1 (8.9–16.8) vs. 8.8 (7.2–11.6), *p* < 0.001], neutrophil counts [9.4 (7.1–12.9) vs. 6.6 (5.0–9.1), *p* < 0.001], CRP levels [30.2 (14.9–115.8) vs. 18.9 (9.0–44.7), *p* = 0.021], blood urea nitrogen (BUN) levels [37.5 (29.7–60.0) vs. 33.0 (22.0–47.7), *p* = 0.039], AST levels [59.0 (27.5–138.5) vs. 26.0 (19.0–60.2), *p* = 0.003], LDH levels [406 (270.5–547.5) vs. 273 (218.7–369.5), *p* = 0.002], and NT-proBNP levels [20,083 (9075–27,760) vs. 8376 (4223–15,885), *p* = 0.024]. They also had a lower estimated glomerular filtration rate (eGFR) [42.0 (30.0–59.5) vs. 48.0 (34.5–66.0) mL/min/1.73 m^2^, *p* = 0.043] and lower chloride levels [96.0 (91.7–100.0) vs. 99.0 (95.0–102.0) mmol/L, *p* = 0.008]. NT-proBNP measurements were available in 169 of 375 patients, whereas troponin measurements were available in 314 of 375 patients.

Regarding hemodynamic parameters, the progression group had lower DBP [60.5 (46.7–70.5) vs. 68.0 (60.0–77.0) mmHg, *p* = 0.007] and higher heart rate [90.5 (82.2–106.0) vs. 78.0 (69.0–90.0) beats/min, *p* < 0.001]. SBP was numerically lower in the progression group, but the difference was not statistically significant (*p* = 0.097). The initial inotropic agent did not differ between groups (*p* = 0.896).

COPD was associated with higher odds of progression in univariable analysis. WBC, CRP, ln(AST), ln(LDH), ln(NT-proBNP), DBP, and heart rate were also significantly associated with progression to SCAI stage C–E (Table 2). Chloride and eGFR were not significantly associated with the outcome. Although chloride and eGFR differed significantly between the groups in unadjusted distributional comparisons, neither variable showed a significant linear association with the odds of progression when analyzed as a continuous predictor in univariable logistic regression. Spearman correlation analysis showed a weak-to-moderate correlation between CRP and WBC (ρ = 0.338, *p* < 0.001), a moderate correlation between AST and LDH (ρ = 0.530, *p* < 0.001), and a very weak correlation between DBP and heart rate (ρ = 0.139, *p* = 0.012).

In the multivariable logistic regression model, higher WBC was independently associated with greater odds of progression to SCAI stage C–E (OR: 1.099, 95% CI: 1.030–1.174, *p* = 0.005), whereas higher DBP was independently associated with lower odds of progression (OR: 0.963 per 1-mmHg increase, 95% CI: 0.934–0.992, *p* = 0.014) (Table 3). In 2000 bootstrap resamples, the association remained significant for WBC (bootstrap *p* = 0.009), whereas the association with DBP was borderline (bootstrap *p* = 0.051).

**Figure 2:**
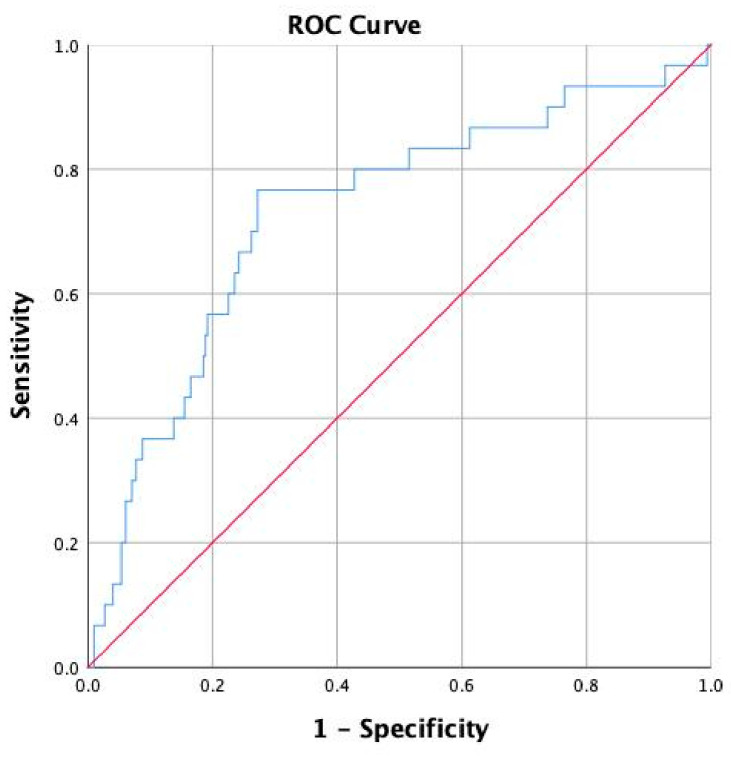
Receiver operating characteristic curve of the multivariable logistic regression model for progression to SCAI stage C–E at 72 h after initiation of inotropic therapy. The area under the curve was 0.731 (95% CI: 0.630–0.832; *p* < 0.001). The blue line represents the receiver operating characteristic curve, and the red dashed diagonal line represents the reference line of no discrimination.

The model showed acceptable calibration according to the Hosmer–Lemeshow test (χ^2^ = 6.987, df = 8, *p* = 0.538), with a Nagelkerke R^2^ of 0.098. The multivariable model showed moderate discrimination, with a c-statistic of 0.731 (95% CI: 0.630–0.832; *p* < 0.001)**,** as illustrated in Figure 2.

## 4. Discussion

In this study, higher WBC and lower DBP were independently associated with progression from SCAI stage A or B to SCAI stage C–E at 72 h after initiation of inotropic therapy in patients with ADHF. Progression occurred in 30 patients (8.0%). The association with WBC remained significant in bootstrap analysis, whereas the association with DBP was borderline, indicating greater uncertainty regarding the stability of the DBP estimate. Accordingly, these findings should be interpreted as exploratory and hypothesis-generating rather than as evidence of robust bedside markers.

Higher WBC was independently associated with progression to SCAI stage C–E, and this association remained significant in bootstrap analysis, supporting the relative stability of the estimate within the present dataset. Neutrophil count and CRP levels were also higher among patients who progressed, suggesting that early hemodynamic deterioration may occur in the context of a broader inflammatory and physiological stress profile. Taken together, these findings support a possible role for systemic inflammatory activation in patients at risk of progression, although they do not establish a causal relationship.

Consistent with our findings, previous studies in patients hospitalized with ADHF have reported associations between higher leukocyte and neutrophil counts, elevated CRP levels, and adverse in-hospital outcomes [10]. However, the prognostic evidence regarding total WBC has not been entirely consistent. In a large AHF cohort, neutrophil count and other leukocyte-derived indices remained associated with mortality, whereas total WBC did not retain an independent association after adjustment for NT-proBNP [11]. Differences in patient selection, outcome definitions, timing of laboratory measurements, and the prevalence of concurrent inflammatory conditions may partly explain these discrepancies. Importantly, the present study evaluated early progression in SCAI stage rather than mortality or rehospitalization. Thus, the observed association suggests that inflammatory activation may be relevant during a distinct and earlier phase of hemodynamic deterioration.

Several mechanisms may underlie the observed association. In AHF, systemic inflammatory activation may contribute to endothelial and microvascular dysfunction and impaired vascular regulation, potentially increasing vulnerability to hemodynamic deterioration [12,13]. Nevertheless, WBC is a nonspecific marker and may be influenced by comorbidities, medications, occult infection, tissue injury, and other stress-related conditions. Although patients with active infection or septic shock were excluded, residual confounding by unrecognized inflammatory states cannot be eliminated. Accordingly, the association between WBC and SCAI progression should be interpreted as prognostic rather than causal, and WBC should not currently be regarded as a validated standalone risk marker.

Clinical presentation may vary considerably among patients with ADHF developing on the background of chronic HF. In patients exposed to long-standing reductions in cardiac output and systemic perfusion, compensatory mechanisms may temporarily maintain blood pressure and end-organ perfusion despite limited hemodynamic reserve [14,15]. This condition may result in a more fragile hemodynamic profile than expected, even without clinically evident signs of hypoperfusion. Previous studies have shown that a substantial proportion of patients classified as SCAI stage B may progress to more advanced stages of cardiogenic shock within the first 24 h [16]. In addition, worsening HF has been reported to be associated with adverse prognosis regardless of its timing and the treatments administered [17]. Therefore, the primary endpoint was defined as progression from baseline SCAI stage A or B to SCAI stage C–E at 72 h, focusing on clinically meaningful deterioration beyond the pre-shock spectrum; however, the observed associations should be interpreted in light of their sensitivity to endpoint specification.

In our study, lower DBP was independently associated with progression to SCAI stage C–E, which may reflect impaired vascular compensation and limited hemodynamic reserve despite the absence of overt hypoperfusion at baseline. In ADHF, DBP may reflect arterial vascular tone, coronary perfusion pressure, and the ability to preserve organ perfusion during clinical stress. Although heart rate was higher among patients who progressed and was associated with progression in univariable analysis, it was not included in the parsimonious multivariable model, in which DBP was selected to represent the hemodynamic domain. COPD was also more frequent among patients who progressed; however, given the limited number of events and the potential for residual confounding, this finding should be interpreted cautiously. Because the bootstrap stability of the DBP association was borderline, this finding should also be interpreted cautiously and not regarded as a robust standalone marker.

Kapur et al. reported that lower SBP was associated with more advanced SCAI stages and higher in-hospital mortality, with early progression occurring even among patients initially classified at lower stages [18]. In our cohort, SBP was not significantly associated with progression, whereas DBP remained significant in the multivariable model. This finding suggests that DBP may provide complementary information regarding early hemodynamic vulnerability; however, the present results do not establish its superiority over SBP.

Previous studies have also suggested prognostic relevance of diastolic pressure-related measures across the heart failure–cardiogenic shock continuum. Low DBP was associated with HF readmission in patients with acute decompensated HFpEF, whereas lower diastolic perfusion pressure was linked to poorer hemodynamic response in established cardiogenic shock [19,20]. Although our cohort differed by including patients without overt hypoperfusion at baseline, these findings collectively suggest that diastolic pressure may provide complementary information regarding circulatory reserve. Nevertheless, given the borderline bootstrap stability and potential construct overlap with SCAI staging, the DBP association should be interpreted cautiously.

NT-proBNP was also associated with progression to SCAI stage C–E in univariable analysis, consistent with its established role as a marker of HF severity and hemodynamic burden. However, NT-proBNP measurements were available only in a subset of the cohort and the variable was therefore not included in the primary multivariable model. Accordingly, its independent association with progression could not be reliably assessed in the primary analysis. The univariable finding should therefore be interpreted cautiously and may primarily reflect the overall severity of cardiac decompensation and hemodynamic burden.

One notable finding was that the distribution of the initial inotropic agent did not differ between patients who progressed to SCAI stage C–E and those who did not. Previous studies comparing levosimendan and dobutamine have suggested that levosimendan may provide more favorable short-term hemodynamic effects; however, evidence regarding clinical outcomes remains inconsistent. In the LIDO trial, levosimendan was associated with a greater hemodynamic response and lower mortality than dobutamine, whereas the larger SURVIVE trial demonstrated no significant difference in long-term mortality between the two agents [21,22]. In our cohort, the absence of a between-group difference should be interpreted cautiously because treatment allocation was not randomized and was based on the attending physician’s clinical judgment. Therefore, our findings do not establish therapeutic equivalence between levosimendan and dobutamine; rather, they indicate that the initial inotropic agent alone did not explain the observed differences in early SCAI stage progression.

From a clinical perspective, WBC may serve as a readily available marker of the inflammatory activation and physiological stress accompanying early hemodynamic deterioration in patients with ADHF receiving intravenous inotropic therapy. The concomitant elevation of neutrophil count and CRP levels among patients who progressed suggests that this association may form part of a broader inflammatory profile. However, WBC is a nonspecific marker and may be influenced by comorbid conditions, medications, tissue injury, and clinically unrecognized inflammatory states. Therefore, the observed association should be interpreted as a prognostic signal accompanying early clinical deterioration rather than as evidence of a causal mechanism.

DBP may provide complementary information regarding hemodynamic reserve, reflecting a different pathophysiological domain from inflammatory stress. Heart rate was associated with progression in univariable analysis but was not included in the parsimonious multivariable model, in which DBP was selected to represent the hemodynamic domain. Accordingly, WBC and DBP should not be regarded as validated standalone risk markers, but rather as complementary clinical variables to be interpreted alongside serial SCAI staging, perfusion findings, inflammatory indices, heart rate, and evolving organ function.

This study has several important limitations. First, its retrospective, single-center design may have introduced selection bias and limits the generalizability of the findings. The decisions to initiate and escalate inotropic or vasopressor therapy were neither protocolized nor randomized and were based on the attending physician’s clinical judgment, creating the possibility of confounding by indication. In addition, owing to the retrospective nature of the records, the interval between hospital admission and initiation of inotropic therapy could not be determined reliably and consistently for all patients and therefore could not be reported quantitatively. Progression to SCAI stage C–E is partly defined by treatment escalation and hemodynamic variables, including blood pressure; therefore, the primary endpoint may reflect both clinical deterioration and treatment decisions, and some degree of construct overlap between the endpoint and DBP cannot be excluded. Accordingly, the observed associations should be interpreted as prognostic rather than causal.

Second, only 30 patients experienced the primary endpoint, which limited the number of variables that could be included in the multivariable model and increased the risk of model instability and overfitting. Accordingly, a parsimonious model was used, and coefficient stability was assessed using bootstrap resampling; however, external validation was not performed. The primary model included 327 patients with complete WBC and DBP data and all 30 outcome events. Although bootstrap resampling supported the stability of the WBC association, the DBP association was borderline, indicating uncertainty regarding the robustness of the DBP estimate. Moreover, the variables associated with adverse hemodynamic progression changed after refinement of the endpoint definition, demonstrating that the findings were sensitive to endpoint specification. This sensitivity should be considered when interpreting the results, and neither WBC nor DBP should be regarded as a validated standalone risk marker.

Third, missing data were present for several variables, particularly NT-proBNP, which was available in only 169 of 375 patients. No imputation was undertaken, and analyses were therefore based on available cases, which may have reduced statistical power and introduced bias if the data were not missing at random. Because of its limited availability, NT-proBNP was not included in the primary multivariable model, and its independent association with progression could not be reliably assessed. Furthermore, lactate measurements and invasive hemodynamic parameters were not uniformly available, limiting a more detailed and objective assessment of tissue hypoperfusion and circulatory status. The potential effects of fluid overload and hemodilution on baseline laboratory measurements could not be standardized or formally adjusted because of the retrospective design. The underlying etiology and World Health Organization classification of pulmonary hypertension in patients with pulmonary hypertension-associated isolated right-sided HF could not be reliably determined from the retrospective records. Finally, the predominance of levosimendan and center-specific treatment practices may further limit applicability to other settings. Despite these limitations, the study provides exploratory information regarding baseline factors associated with early progression to advanced SCAI stages in a selected high-risk population with ADHF receiving inotropic therapy. These findings require confirmation in larger, prospective, multicenter cohorts.

## 5. Conclusions

In patients with ADHF who were classified as SCAI stage A or B at baseline and received intravenous inotropic therapy, higher WBC and lower DBP were associated with progression to SCAI stage C–E at 72 h. Given the limited number of events, the sensitivity of the findings to endpoint specification, and the borderline bootstrap stability of the DBP association, these results should be considered exploratory and hypothesis-generating. Serial assessment and repeated SCAI staging may help identify patients at risk of early deterioration. Prospective multicenter studies are needed to validate these findings.

## Figures and Tables

**Table 1:** Baseline Clinical, Echocardiographic, Laboratory, Hemodynamic, and Treatment Characteristics According to Progression to SCAI Stage C–E at 72 Hours.

Variable	Overall (*n* = 375)	Progression to SCAI Stage C–E (*n* = 30)	No Progression to SCAI Stage C–E (*n* = 345)	*p*-Value
**Demographics and comorbidities**				
Age, years	69 (60–79)	71 (64–78)	69 (60–79)	0.451
Male sex, *n* (%)	278 (74.1)	21 (70.0)	257 (74.5)	0.590
Hypertension, *n* (%)	243 (65.0)	15 (50.0)	228 (66.3)	0.073
Diabetes mellitus, *n* (%)	165 (44.1)	10 (33.3)	155 (45.1)	0.215
COPD, *n* (%)	39 (10.5)	8 (26.7)	31 (9.0)	0.007
CKD, *n* (%)	144 (38.4)	12 (40.0)	132 (38.3)	0.851
ICD, *n* (%)	56 (15.0)	1 (3.3)	55 (16.0)	0.256
Heart failure etiology, *n* (%)				0.100
Ischemic	236 (62.9)	18 (60)	218 (63.2)	
Non-ischemic	115 (30.7)	9 (30)	106 (30.7)	
Unknown	24 (6.4)	3 (10)	21 (6.1)	
Heart failure phenotype, *n* (%)				0.409
HFrEF	338 (90.1)	29 (96.7)	309 (89.6)	
HFmrEF	23 (6.1)	1 (3.3)	22 (6.4)	
Pulmonary hypertension-associated isolated right-sided heart failure	14 (3.7)	0 (0.0)	14 (4.1)	
**Echocardiographic findings**				
LVEF, %	25 (20–30)	25 (15–30)	25 (20–30)	0.365
LVEDD, mm	59.3 ± 8.1	59.4 ± 8.6	59.4 ± 8.1	0.982
LVESD, mm	49 (44–55)	50 (42–56)	49 (44–55)	0.087
IVS thickness, mm	11 (10–12)	10 (9–12)	11 (10–12)	0.130
Posterior wall thickness, mm	10 (10–12)	10 (9.5–11)	10 (10–12)	0.155
LA diameter, mm	48 (44–51)	45 (42.5–50.5)	48 (44–51)	0.092
PASP, mmHg	43 (35–56)	41 (33.5–55.5)	43 (35–56)	0.611
Significant valvular disease, *n* (%)	256 (68.4)	18 (60.0)	238 (69.2)	0.299
Severe mitral regurgitation, *n* (%)	196 (52.9)	17 (58.6)	179 (52.5)	0.610
Severe aortic regurgitation, *n* (%)	14 (3.8)	0 (0.0)	14 (4.1)	0.305
Severe tricuspid regurgitation, *n* (%)	194 (52.4)	12 (41.3)	182 (53.4)	0.246
Severe mitral and tricuspid regurgitation, *n* (%)	137 (37.0)	11 (37.9)	126 (36.9)	0.987
**Laboratory findings**				
Hemoglobin, g/dL	11.7 (10.4–13.4)	11.9 (10.6–13.4)	11.7 (10.4–13.5)	0.173
WBC, ×10^9^/L	8.95 (7.3–11.7)	11.1 (8.9–16.8)	8.8 (7.2–11.6)	<0.001
Neutrophil count, ×10^9^/L	6.8 (5.1–9.3)	9.4 (7.1–12.9)	6.6 (5.0–9.1)	<0.001
Lymphocyte count, ×10^9^/L	1.1 (0.7–1.6)	1.3 (0.9–1.9)	1.10 (0.7–1.6)	0.275
CRP, mg/L	19.8 (9.2–46.0)	30.2 (14.9–115.8)	18.9 (9.0–44.7)	0.021
BUN, mg/dL	33 (22–48)	37.5 (29.7–60)	33 (22–47.7)	0.039
eGFR, mL/min/1.73 m^2^	46.5 (34–64)	42 (30–59.5)	48 (34.5–66)	0.043
Creatinine, mg/dL	1.44 (1.1–1.8)	1.6 (1.1–2.2)	1.4 (1.1–1.8)	0.135
ALT, U/L	23 (13.7–56.2)	39 (14.7–140.5)	23 (13–51.5)	0.035
AST, U/L	27 (19–68)	59 (27.5–138.5)	26 (19–60.2)	0.003
LDH, U/L	278 (224–388.7)	406 (270.5–547.5)	273 (218.7–369.5)	0.002
NT-proBNP, pg/mL	8613 (4412–16,083)	20,083 (9075–27,760)	8376 (4223–15,885)	0.024
Troponin, ng/L	56 (29–208)	131 (35–1580)	54 (28–190.5)	0.067
Sodium, mmol/L	136 (132–139)	134.5 (132–138)	136 (132–139)	0.405
Potassium, mmol/L	4.4 (3.9–4.8)	4.6 (3.8–4.8)	4.3 (3.9–4.8)	0.979
Chloride, mmol/L	99 (95–102)	96 (91.7–100)	99 (95–102)	0.008
Magnesium, mg/dL	2.0 (1.8–2.2)	2.0 (1.7–2.2)	2.0 (1.8–2.2)	0.510
Albumin, g/L	36.4 (33–39.6)	35.4 (31.7–38)	36.7 (33–39.8)	0.103
**Hemodynamic and rhythm variables**				
SBP, mmHg	110 (100–122)	104 (92–119.2)	110 (100–122)	0.097
DBP, mmHg	67 (60–76)	60.5 (46.7–70.5)	68 (60–77)	0.007
Heart rate, bpm	80 (70–91)	90.5 (82.2–106)	78 (69–90)	<0.001
Atrial fibrillation, *n* (%)	68 (18.1)	3 (10.0)	65 (18.8)	0.843
**Initial inotropic treatment**				
Levosimendan, *n* (%)	254 (67.7)	20 (66.7)	234 (67.8)	0.896
Dobutamine, *n* (%)	121 (32.3)	10 (33.3)	111 (32.2)	
**Additional vasoactive treatment after baseline**				
Multiple inotropes and/or vasopressors, *n* (%)	52 (13.9)	22 (73.3)	30 (8.7)	<0.001
At least two inotropes	15 (4.0)	2 (6.7)	13 (3.8)	
One inotrope plus norepinephrine	17 (4.5)	10 (33.3)	7 (2.0)	
At least two inotropes plus norepinephrine	20 (5.3)	10 (33.3)	10 (2.9)	
**In-hospital outcome**				
In-hospital mortality, *n* (%)	60 (16.0)	15 (50.0)	45 (13.0)	<0.001

**Note:** Data are presented as mean ± standard deviation, median (interquartile range), or n (%), as appropriate. For variables with missing data, percentages were calculated using the number of patients with available data for the corresponding variable as the denominator. NT-proBNP and troponin measurements were available for 169 and 314 patients, respectively. Heart failure etiology was classified separately as ischemic, non-ischemic, or unknown according to the available clinical records. The patient counts and percentages for valvular disease subcategories were calculated using the number of patients with available valvular data in each group. These subcategories were not mutually exclusive because individual patients could have more than one valvular lesion. Post-baseline vasoactive treatment subcategories were mutually exclusive and summed to the corresponding parent category. Post-baseline vasoactive treatments and in-hospital mortality are presented descriptively and were not included as baseline predictors in the multivariable model. All in-hospital deaths occurred after the 72 h SCAI assessment. Abbreviations: ALT, alanine aminotransferase; AST, aspartate aminotransferase; BUN, blood urea nitrogen; CKD, chronic kidney disease; COPD, chronic obstructive pulmonary disease; CRP, C-reactive protein; eGFR, estimated glomerular filtration rate; HF, heart failure; HFmrEF, heart failure with mildly reduced ejection fraction; HFrEF, heart failure with reduced ejection fraction; ICD, implantable cardioverter-defibrillator; IVS, interventricular septum; LA, left atrium; LDH, lactate dehydrogenase; LVEDD, left ventricular end-diastolic diameter; LVEF, left ventricular ejection fraction; LVESD, left ventricular end-systolic diameter; NT-proBNP, N-terminal pro-B-type natriuretic peptide; PASP, pulmonary artery systolic pressure; SCAI, Society for Cardiovascular Angiography and Interventions; WBC, white blood cell count; SBP, systolic blood pressure; DBP, diastolic blood pressure.

**Table 2:** Univariable Logistic Regression Analysis for Progression to SCAI Stage C–E at 72 Hours After Initiation of Inotropic Therapy.

Variable	OR	95% CI	*p*-Value
WBC	1.108	1.040–1.179	0.001
C-reactive protein	1.008	1.003–1.013	0.002
ln(AST)	1.355	1.031–1.780	0.029
ln(LDH)	1.939	1.138–3.303	0.015
ln(NT-proBNP)	3.065	1.079–8.707	0.035
DBP	0.959	0.931–0.989	0.007
Heart rate	1.034	1.015–1.054	0.001
Chloride	0.996	0.974–1.020	0.759
eGFR	0.991	0.974–1.008	0.294
COPD	3.660	1.504–8.907	0.004

Abbreviations: AST, aspartate aminotransferase; CI, confidence interval; COPD, chronic obstructive pulmonary disease; CRP, C-reactive protein; eGFR, estimated glomerular filtration rate; LDH, lactate dehydrogenase; ln, natural logarithm; NT-proBNP, N-terminal pro-B-type natriuretic peptide; OR, odds ratio; WBC, white blood cell count; DBP, diastolic blood pressure.

**Table 3:** Multivariable Logistic Regression Analysis of Factors Associated with Progression to SCAI Stage C–E at 72 h After Initiation of Inotropic Therapy.

Variable	OR	95% CI	*p*-Value
WBC	1.099	1.030–1.174	0.005
DBP	0.963	0.934–0.992	0.014

Note: The primary multivariable model included 327 patients and 30 outcome events. Model calibration: Hosmer–Lemeshow χ^2^ = 6.987, df = 8, *p* = 0.538. Model explanatory value: Nagelkerke R^2^ = 0.098. Model discrimination: c-statistic = 0.731 (95% CI: 0.630–0.832; *p* < 0.001). In 2000 bootstrap resamples, the association remained significant for WBC (bootstrap *p* = 0.009), whereas the association with DBP was borderline (bootstrap *p* = 0.051). Abbreviations: CI, confidence interval; OR, odds ratio; SCAI, Society for Cardiovascular Angiography and Interventions; WBC, white blood cell count; DBP, diastolic blood pressure.

## Data Availability

The data sets created and/or analyzed during the current study are not publicly available because they contain patient information, but data supporting the findings of this study can be obtained from the corresponding author (RA) upon reasonable request.

## Abbreviations

The following abbreviations are used in this manuscript:

ADHF	Acute decompensated heart failure
AHF	Acute heart failure
ALT	Alanine aminotransferase
AST	Aspartate aminotransferase
BUN	Blood urea nitrogen
CI	Confidence interval
CICU	Coronary intensive care unit
CKD	Chronic kidney disease
COPD	Chronic obstructive pulmonary disease
CRP	C-reactive protein
DBP	Diastolic blood pressure
EF	Ejection fraction
eGFR	Estimated glomerular filtration rate
HF	Heart failure
HFmrEF	Heart failure with mildly reduced ejection fraction
HFpEF	Heart failure with preserved ejection fraction
HFrEF	Heart failure with reduced ejection fraction
ICD	Implantable cardioverter-defibrillator
IQR	Interquartile range
IVS	Interventricular septum
LA	Left atrium
LDH	Lactate dehydrogenase
LVEDD	Left ventricular end-diastolic diameter
LVEF	Left ventricular ejection fraction
LVESD	Left ventricular end-systolic diameter
NT-proBNP	N-terminal pro-B-type natriuretic peptide
OR	Odds ratio
PASP	Pulmonary artery systolic pressure
SCAI	Society for Cardiovascular Angiography and Interventions
SBP	Systolic blood pressure
WBC	White blood cell count
